# Higher Handgrip Strength Is Linked to Better Cognitive Performance in Chinese Adults with Hypertension

**DOI:** 10.3390/brainsci11080985

**Published:** 2021-07-25

**Authors:** Shenghua Lu, Fabian Herold, Yanjie Zhang, Yuruo Lei, Arthur F. Kramer, Can Jiao, Qian Yu, Scott Doig, Jinming Li, Zhe Yan, Jin Kuang, Ting Wang, Liye Zou

**Affiliations:** 1 Hunan Academy of Education Sciences, Changsha 225002, China; lushenghua6666@126.com; 2 College of Sports Science, Jishou University, Jishou 416000, China; 3 Research Group Neuroprotection, German Center for Neurodegenerative Diseases (DZNE), Leipziger Str. 44, 39120 Magdeburg, Germany; fabian.herold@st.ovgu.de; 4 Department of Neurology, Medical Faculty, Otto von Guericke University, Leipziger Str. 44, 39120 Magdeburg, Germany; 5 Health and Exercise Science Laboratory, Institute of Sports Science, Seoul National University, Seoul 08826, Korea; 6 Physical Education Unit, School of Humanities and Social Science, The Chinese University of Hong Kong-Shenzhen, Shenzhen 518172, China; 7 Institute of Urban Governance, Shenzhen University, Shenzhen 518060, China; 8 Center for Cognitive and Brain Health, Northeastern University, Boston, MA 02115, USA; a.kramer@northeastern.edu; 9 Beckman Institute, University of Illinois at Urbana-Champaign, Champaign, IL 61801, USA; 10 Institute of KEEP Collaborative Innovation, Shenzhen University, Shenzhen 518060, China; jiaocan@szu.edu.cn (C.J.); yuqianmiss@163.com (Q.Y.); jinmingli1999@gmail.com (J.L.); zoeyan0610@163.com (Z.Y.); dennyppg89@gmail.com (J.K.); janewang10142021@163.com (T.W.); liyezou123@gmail.com (L.Z.); 11 Exercise Psychophysiology Laboratory, School of Psychology, Shenzhen University, Shenzhen 518060, China; 12 Department of Physical Education, Limestone University, Gaffney, SC 29340, USA; srdoig@limestone.edu

**Keywords:** handgrip strength, cognition, hypertension, China

## Abstract

Objective: There is growing evidence that in adults, higher levels of handgrip strength (HGS) are linked to better cognitive performance. However, the relationship between HGS and cognitive performance has not been sufficiently investigated in special cohorts, such as individuals with hypertension who have an intrinsically higher risk of cognitive decline. Thus, the purpose of this study was to examine the relationship between HGS and cognitive performance in adults with hypertension using data from the Global Ageing and Adult Health Survey (SAGE). Methods: A total of 4486 Chinese adults with hypertension from the SAGE were included in this study. Absolute handgrip strength (aHGS in kilograms) was measured using a handheld electronic dynamometer, and cognitive performance was assessed in the domains of short-term memory, delayed memory, and language ability. Multiple linear regression models were fitted to examine the association between relative handgrip strength (rHGS; aHGS divided by body mass index) and measures of cognitive performance. Results: Overall, higher levels of rHGS were associated with higher scores in short-term memory (β = 0.20) and language (β = 0.63) compared with the lowest tertiles of rHGS. In male participants, higher HGS was associated with higher scores in short-term memory (β = 0.31), language (β = 0.64), and delayed memory (β = 0.22). There were no associations between rHGS and cognitive performance measures in females. Conclusion: We observed that a higher level of rHGS was associated with better cognitive performance among hypertensive male individuals. Further studies are needed to investigate the neurobiological mechanisms, including sex-specific differences driving the relationship between measures of HGS and cognitive performance in individuals with hypertension.

## 1. Introduction

Hypertension is a major cause of cardiovascular diseases (e.g., stroke), affecting about 40% of the aging population globally [1]. The increasing incidence of hypertension characterized by high blood pressure has become an urgent public health problem, and nearly 60% of Chinese older adults are suffering from hypertension [2]. In this context, it is worth noting that hypertension can increase the risk of cognitive decline since it affects the brain negatively [3,4,5,6], although the neurobiological mechanisms driving the relationship between hypertension and cognitive performance are controversially discussed and not fully understood. In a study by Tzourio et al., a non-significant association between hypertension and cognitive performance was observed [7], whereas other studies reported that hypertension (i) was associated with structural brain damage, (ii) was associated with a deficit in cognitive performance, and (iii) could accelerate the development of dementia [3,5,8,9]. Moreover, cognitive impairments can affect individuals’ mental health and social functioning and even cause an increase in the economic burden to take care of individual symptoms caused by the disease [10]. Notably, it has also been observed that individuals with hypertension who meet established physical activity guidelines showed superior cognitive performance [11], thus buttressing the claim that regular physical exercises are important to ensure brain health and cognition in adults with hypertension [12]. Collectively, the available evidence shows that hypertension has a detrimental effect on cognitive health, and thus identifying risk and protective factors before a manifestation of cognitive impairment occurs is essential in individuals with hypertension.

Handgrip strength (HGS), an important indicator of physical function, has been linked to various physical health outcomes (e.g., increased risk of falls, depression, and pre-mortality) among older adults [13,14,15,16]. Moreover, there is mounting evidence that measures of HGS are associated with cognitive health [17,18,19]. In particular, previous studies have established a relationship between measures of HGS and cognitive performance in younger adults [20], middle-aged adults [21], and older adults [22,23]. For example, a longitudinal study of older adults indicated that the participants in the highest quartile of rHGS had a 50% lower risk to develop cognitive impairments compared with participants in the lowest quartile of rHGS [24]. In line with this finding, a cross-sectional study of obese females reported that individuals in the lowest tertile of aHGS had 84% higher odds for cognitive impairments compared with obese females in the highest tertile of aHGS [25]. In this context, there is also emerging evidence that biological sex could be an important moderator in the relationship between physical fitness and cognitive performance [26,27,28,29]. However, sex-specific effects in the relationships of HGS and cognitive performance have not been extensively investigated, even though there is some evidence that the association between HGS and prevalence of mild cognitive impairments is more pronounced in men [28]. Collectively, these findings imply that individuals with lower measures of HGS may have a higher risk to develop cognitive impairments and that this relationship is moderated by biological sex.

Despite the fact that the above-mentioned studies have shown that measures of HGS can be important markers to identify adults at higher risk for cognitive decline, it has not been sufficiently studied whether this finding can be generalized to an adult population with hypertension. Adults with hypertension are intrinsically at a higher risk to develop cognitive impairments (e.g., due to hypertension-related brain changes). To address this gap in the literature, this study investigated whether rHGS is associated with performance in specific cognitive subdomains in both male and female adults with hypertension. Thus, the current study will add novel evidence to the literature, as it is among the first studies that (i) investigated the relationship between rHGS and performance in specific cognitive domains in hypertensive adults and (ii) examined the moderating role of biological sex in the relationship between rHGS and cognitive performance. Based on the available evidence, we hypothesized that in adults with hypertension, higher levels of rHGS are associated with better cognitive performance.

## 2. Materials and Methods

### 2.1. Study Population

This cross-sectional study used publicly available data from the Global Ageing and Adult Health Survey (SAGE: http://www.who.int/healthinfo/sage/en accessed on 29 March 2021). This survey assessed data of a nationally representative sample of Chinese individuals and was carried out between 2007 and 2010 across eight provinces. All procedures of data collection were reported in a previous study in more detail [30]. In brief, well-trained interviewers conducted face-to-face household interview surveys (questionnaire) to collect the data. The survey response rate was 93%. The study was approved by the World Health Organization Ethical Review Committee and the Chinese Ethics Research Review Board of Peking University (IRB00001052-11014 and IRB00001052-11015). Each participant signed informed written consent before she/he participated in the experiment.

Data were extracted on demographic characteristics (e.g., age and sex), handgrip strength, cognitive performance, and diagnosed hypertension from the Chinese cohort of SAGE. All individuals without hypertension were excluded and were not considered in the data analysis. As a result, a total of 4486 individuals with hypertension were included for analysis in this study.

### 2.2. Hypertension Diagnosis

Participants were defined as hypertensive if they selected “Yes” on “Have you ever been diagnosed with high blood pressure (hypertension)?” or if they had high blood pressure values, which were systolic blood pressure (SBP) ≥140 mmHg and/or diastolic blood pressure (DBP) ≥90 mm Hg [31].

### 2.3. Handgrip Strength

Absolute handgrip strength (aHGS) in kilograms (kg) was measured by trained assessors using a Smedley’s hand dynamometer (Scandidact, Oldenvej 45, and 3490 Kvistgard, Denmark) [32]. The participants were seated in a comfortable chair with their feet flat on the ground. They were asked to keep their upper arm against their body and to bend their elbow to 90 degrees with their palm facing in (as if shaking hands). The trained assessors instructed the seated participants to hold the dynamometer. The participants were asked to squeeze the hand as hard as they could, and each hand was tested twice. The overall HGS was the average of the best aHGS for each hand. If the participants had any surgery on their hand, arm, or wrist in the past 3 months or arthritis or pain in the hand/wrist/arm, aHGS was not assessed for that hand. As previously described [33], we calculated relative handgrip strength (rHGS) by dividing overall HGS (i.e., the average of the best aHGS) by body mass index (BMI).

### 2.4. Cognitive Performance

Cognitive performance was assessed using (i) the Digit Span Forward and Backward Test, (ii) the Verbal Fluency Test, and (iii) the Verbal Recall Test.

The Digit Span Forward (DSF) and Digit Span Backward (DSB) tests were used to quantify short-term memory and working memory performance. The participants were required to repeat a set of orally presented items (i.e., numbers) while the level of difficulty was gradually increased by one item for each trial. Both the DSF and the DSB start with a sequence of two numbers (e.g., 9 − 5), and for each span, two trials are performed. For each trial, a new sequence of items was presented. The researcher stopped the test after two consecutive incorrect responses on the same set of items [34]. Performance was scored based on the maximum length of correctly remembered items. The highest achievable scores for the DSF and DSB were 9 points and 8 points, respectively.

The semantic Verbal Fluency Test (VFT) probes language abilities (i.e., lexical retrieval and production). Participants were required to name as many animals as they could within one minute. One point was given for a correctly named animal (nonrepeating) [35].

The Verbal Recall Test (VRT) was used to measure performance in delayed memory. An interviewer read 10 words for participants three times at a standardized pace. After a delay of 10 min, the participants were required to recall as many words as possible from the initially presented 10 words. One point was given for each correctly recalled word [36].

### 2.5. Independent Variables

Age, sex, years of education, setting (rural or urban), alcohol consumption in the past month, smoking (never, current, or past), chronic physical conditions (stroke and diabetes), and regular level of physical activity (PA; occupational and leisure PA) [37] were used as covariates in the statistical models. Please note that the classifications of stroke and diabetes were based on self-reports of existing diagnoses from medical professionals. In line with a previous study, PA was measured using the Global Physical Activity Questionnaire Version 2 (GPAQ-V2), which consists of 16 questions [38]. The GPAQ-V2, which was developed under the auspices of the World Health Organization (WHO), is an internationally recognized, reliable, and validated questionnaire that assesses levels of regular physical activity [39,40]. According to the PA calculation from a previous study [37], levels of physical activity were classified as ≥150 min/week (meeting the recommended guidelines) and <150 min/week (low PA).

### 2.6. Statistical Analysis

In this study, continuous variables are expressed as means and standard deviation (SD), whereas categorical variables are shown as percentages. On the basis of the known sex difference in aHGS and cognitive performance in later life [22], we analyzed males and females separately (see Table 1, Table 2 and Table 3). Multivariable linear regression models were used to estimate the associations between rHGS and cognitive performance (Digit Span Forward and Backward Test, Verbal Fluency Test, and Verbal Recall Test). As the interaction terms of rHGS and sex in our linear regression showed a significant difference in all cognitive performance tests (*p* < 0.05), we estimated the associations between rHGS and cognitive performance (Digit Span Forward and Backward Test, Verbal Fluency Test, and Verbal Recall Test) and adjusted the regression models for age, sex, education years, setting, alcohol consumption, smoking, chronic physical conditions, and physical activity. All statistical analyses were conducted using the Stata 15.0 (Stata Corp LP, College Station, Texas). The level of statistical significance for all statistical tests was set at *p* < 0.05 (two-tailed).

## 3. Results

The final sample included in this study consisted of 4486 adults with hypertension. Table 1 provides an overview of the demographic characteristics, rHGS, and cognitive performance. The mean age for the sample was 59.68 ± 0.19 for male participants and 59.39 ± 0.40 for female participants. Furthermore, our sample included more male participants (80.7%) than female participants (19.3%).

As shown in Table 1, we observed significant differences between male and female participants concerning the level of education, setting, alcohol consumption, smoking, level of regular occupational PA, and performance in DSF, DSB, and VFT (all *p* < 0.05).

Table 2 displays the characteristics of participants according to the tertile of HGS for male and female participants. We observed that age, DSF, DSB, VFT, and VRT varied as a function of the tertile of HGS (all *p* < 0.05).

Table 3 displays the adjusted associations between HGS and cognitive function scores in adults with hypertension. Overall, when controlling for age, sex, level of education, setting, alcohol consumption, smoking, level of regular occupational PA, and leisure PA, individuals in the highest tertile of rHGS performed better in DSF and VFT compared with individuals in the lowest tertile of HGS (*p* < 0.01). Independent associations were found between rHGS and performance in DSF and VRT (*p* < 0.05) for male participants in the tertile of moderate and high rHGS. In the highest tertile of rHGS, rHGS was associated with performance on the VFT (*p* < 0.01).

## 4. Discussion

The current study investigated the associations of rHGS and cognitive performance in a Chinese sample of hypertensive individuals recruited from the SAGE project. Overall, our findings suggest that in adults with hypertension, higher levels of rHGS are associated with better cognitive performance in short-term memory (assessed using the Digit Span Forward and Digit Span Backward tests) and language abilities (assessed using the Verbal Fluency test) after controlling for important covariates (e.g., age, sex). Furthermore, we observed pronounced sex differences, as an association between higher levels of rHGS and better performance on the DSF, VFT, and VRT could be observed in male participants but not in female participants.

Our findings are in line with previous studies showing a relationship between the level of HGS and scores in short-term memory, language, and delayed memory (but not working memory) [18,41]. For example, a scoping review suggested that higher levels of HGS were associated with a lower risk of cognitive impairments in older adults [18]. A prospective study by Alfaro-Acha et al. found that lower aHGS was associated with a deficit in cognitive function in 2160 older Mexican Americans over 7 years [41]. In a cross-sectional study of 449 adults without dementia from Sweden, Praetorius Bjork et al. showed that there are strong relationships between aHGS and short-term memory, delayed memory, and language abilities [42].

Furthermore, the current study substantiates the available evidence regarding the link between measures of HGS and cognitive performance in adults with hypertension. A comparable study observed a link between aHGS and better visuospatial abilities, episodic memory, orientation/attention, and overall cognitive function in a cohort of middle-aged and older adults with hypertension [43]. Thus, our study adds evidence to the current literature that (i) in adults with hypertension, the link between rHGS and cognitive performance does not only comprise visuospatial abilities, episodic memory, orientation/attention, and overall cognitive function but also encompasses short-term memory, delayed memory, and language abilities and (ii) there are pronounced sex-specific differences in the association between rHGS and cognitive performance.

There is some evidence from a cross-sectional study [29] and a meta-analysis of interventional studies [44] that females profit more from physical interventions than males, although this finding is not universal [45]. Even if the mentioned studies are not fully comparable with our study, the direction of the sex-specific differences in our study is somewhat surprising. As suggested by others, this could be caused by sex-specific differences in neurobiological mechanisms driving behavioral performance.

Overall, the findings of the current study suggest that higher levels of rHGS are linked to better cognitive performance, although the neurobiological mechanisms driving this relationship are yet not fully clear and warrant future investigation [46,47,48]. In this context, several possible mechanisms could explain the positive relationship between measures of HGS and cognitive performance. In particular, it has been proposed that the following levels of analysis need to be considered to understand the effect of physical activity or physical fitness (i.e., level of HGS) on cognitive performance: (i) level 1—molecular and cellular changes, (ii) level 2—functional and structural brain changes, and (iii) level 3—socioemotional changes [47,49].

Our knowledge concerning the relationship of measures of HGS and cognition-relevant changes in level 1 (cellular and molecular changes) is meager. With respect to the association between cardiovascular fitness and/or cardiovascular exercise and cognitive performance, brain-derived neurotrophic factor (BDNF) and other neurotrophic factors have been highlighted to play a crucial role [49,50,51]. In this regard, there is some evidence that (i) cardiorespiratory fitness level is associated with basal levels of BDNF [52,53], (ii) the lower levels of serum BDNF are associated with a smaller hippocampal volume and poorer memory performance [54], and (iii) changes in BDNF levels in response to long-term aerobic training are associated with changes in executive functions [55] and hippocampal volume [56]. Collectively, these studies suggest that BDNF plays a crucial role in the relationship between cardiorespiratory fitness and cognitive performance. However, comparable studies investigating the relationship between HGS, neurotrophic factors such as BDNF, and cognitive performance are, to the best of our knowledge, currently lacking. Thus, further studies investigating the relationship between HGS and neurotrophic factors (e.g., BNDF) are needed to elucidate the extent to which changes on this level of analysis can explain some variance in the positive relationship between measures of HGS and cognitive performance.

With respect to level 2 (functional and structural brain changes), it has been proposed that HGS and higher-order cognitive functions might share the same neural substrates [7]. Indeed, there is some evidence in the literature suggesting a close relationship between HGS and brain features relevant to higher-order cognitive processes. Concerning functional brain changes, it has been observed that in younger adults, higher levels of normalized HGS are linked to favorable cerebral hemodynamics [47]. However, in this study, normalized HGS was not linked to cognitive performance, nor did this study find convincing evidence that cortical hemodynamics mediate a possible relationship between normalized HGS and cognitive performance [7]. However, as this finding cannot be readily generalized on the basis of our cohort, additional studies are needed to elucidate whether HGS might influence cortical hemodynamics in adults with hypertension. Regarding structural brain changes, there is evidence in the literature linking higher levels of aHGS to greater hippocampal volume [57]. Given that a greater hippocampal volume [58] and an increase in hippocampal volume [56] are linked to better cognitive performance (i.e., spatial memory), it seems plausible to hypothesize that alterations in brain structure at least partly influence the relationship between higher levels of HGS and cognitive performance. This idea is reinforced by the fact that (i) resistance training improves hippocampal integrity in older adults with mild cognitive impairment [59,60] and (ii) the hippocampus mediates the relationship between higher levels of physical fitness (i.e., cardiorespiratory fitness) and spatial memory performance [56,58]. In this context, it could be speculated that the neurobiological processes leading to a higher HGS might be protective against the frequently reported hypertension-related worsening of brain integrity (e.g., faster decline in hippocampal volume and more global brain matter atrophy) and cognitive performance (e.g., more pronounced decline in executive function and memory) [61,62,63,64]. However, to the best of our knowledge, there is a lack of cross-sectional and longitudinal studies that assess and analyze the relationship between measures of HGS, brain structure, and cognitive performance. Thus, future investigations that provide empirical evidence to verify or refute the above-mentioned theoretical assumptions while considering sex-related differences in structural brain changes (e.g., those reported by intervention studies [44]) are urgently needed.

Level 3 (socioemotional changes) comprises changes in mood, stress, pain, and sleep. It is beyond the scope and intention of this article to discuss the relationship between HGS and these socioemotional factors. However, sleep has been highlighted as an important mediator in the relationship between physical activity and/or physical fitness and cognitive performance [49,65]. It was observed that unhealthy sleep patterns (e.g., too short or too long sleep duration and insufficient sleep quality) can be linked to weaker HGS and faster decline of HGS in adults [66,67,68]. Furthermore, empirical evidence suggests that sleep patterns mediate the relationship between regular physical activity and cognitive performance (e.g., inhibition performance) [69,70]. It seems reasonable to hypothesize that changes in socioemotional factors (e.g., sleep) and their influence on the other levels of analysis (e.g., the cellular and molecular level and the functional and structural level) can at least partly explain the positive relationships between measures of HGS and cognitive performance. However, given the lack of research in this direction, future studies are required to test these assumptions empirically.

### Limitations

The findings of the current study need to be interpreted in light of some limitations. First, this cross-sectional study does not allow the assessment of causal mechanisms driving the relationship between rHGS and cognitive performance. Second, we used only rHGS as a predictor of physical performance, whereas other measures of physical performance (e.g., gait speed and sit-to-stand performance), which have been used in previous studies investigating the relationship between physical fitness and cognitive performance in hypertensive adults [43,71], were not analyzed because of a lack of available data. Third, the data assessment for the SAGE study was carried out between 2007 to 2010, which might affect the generalizability of the findings to a certain extent. However, given the fact that hypertension is still a major health issue, as it constitutes an important risk factor for heart diseases, stroke, chronic kidney disease, and dementia [72,73], we believe that the findings of the current study are still of interest for the scientific community.

## 5. Conclusions

In conclusion, the findings of the current study suggest that a higher level of rHGS is related to better performance in specific domains of cognition (i.e., short-term memory, delayed memory, and language abilities) in our sample of Chinese adults with hypertension (especially in male adults). Further studies are needed to investigate the neurobiological mechanisms driving this relationship, including the identification of sex-specific differences.

## Figures and Tables

**Table 1:** Overview of demographic characteristics and measures of handgrip strength and cognitive performance.

Variables		Male (3620)	Female (866)
Age (in years)		59.68 ± 0.19	59.39 ± 0.40
Education (in years)		7.54 ± 0.07	6.59 ± 0.18
Setting (in %)	Rural	59.01	52.89
	Urban	40.99	47.11
Alcohol consumption (in %)	Yes	70.80	53.00
	No	29.20	47.00
Smoking (in %)	Never	20.75	86.14
	Current	64.71	10.00
	Past	14.54	3.86
Work physical activity (in %)	≤150 min/week	76.24	83.72
	>150 min/week	23.76	16.28
Leisure physical activity (in %)	≤150 min/week	97.29	97.69
	>150 min/week	2.71	2.31
Stroke (in %)	Yes	3.15	2.08
	No	96.85	97.92
Diabetes (in %)	Yes	4.63	4.52
	No	95.37	95.48
Handgrip strength (kg)		33.74 ± 0.18	22.95 ± 0.28
Digit span forward (score)		7.33 ± 0.03	7.21 ± 0.05
Digit span backward (score)		3.67 ± 0.02	3.06 ± 0.05
Verbal fluency (score)		13.82 ± 0.08	12.77 ± 0.15
Delay recall (score)		5.19 ± 0.04	5.13 ± 0.08

**Table 2:** Participants’ characteristics based on the tertiles of handgrip strength in male and female participants.

Variables	*Q*3 (1.579–6.347)	*Q*2 (1.147–1.578)	*Q*1 (0.224–1.146)	*p* Value
**Male**				
Age (*n* = 3421) (in years)	55.46 ± 0.30	60.49 ± 0.29	65.21 ± 0.37	*p* < 0.001
Handgrip strength (kg)	42.46 ± 0.23	32.68 ± 0.14	21.30 ± 0.22	*p* < 0.001
Digit span forward (*n* = 3414) (score)	7.61 ± 0.04	7.37 ± 0.04	6.90 ± 0.05	*p* < 0.001
Digit span backward (*n* = 3406) (score)	3.81 ± 0.04	3.60 ± 0.04	3.50 ± 0.05	*p* < 0.001
Verbal fluency (*n* = 3414) (score)	14.53 ± 0.14	13.80 ± 0.14	12.84 ± 0.17	*p* < 0.001
Delay recall (*n* = 3409) (score)	5.58 ± 0.06	5.22 ± 0.06	4.67 ± 0.08	*p* < 0.001
**Female**				
Age (*n* = 829) (in years)	53.98 ± 1.53	55.22 ± 0.76	61.20 ± 0.47	*p* < 0.001
Handgrip strength (kg)	37.23 ± 1.00	29.23 ± 0.28	19.29 ± 0.25	*p* < 0.001
Digit span forward (*n* = 829) (score)	7.41 ± 0.19	7.51 ± 0.09	7.13 ± 0.06	*p* = 0.02
Digit span backward (*n* = 827) (score)	3.57 ± 0.15	3.25 ± 0.09	2.96 ± 0.06	*p* < 0.001
Verbal fluency (*n* = 829) (score)	14.05 ± 0.55	13.19 ± 0.32	12.52 ± 0.19	*p* = 0.014
Delay recall (*n* = 825) (score)	6.07 ± 0.19	5.84 ± 0.15	4.86 ± 0.10	*p* < 0.001

Note: Q1, Low tertile of relative handgrip strength (rHGS); Q2, Moderate tertile of relative handgrip strength (rHGS); Q3, High tertile of relative handgrip strength (rHGS).

**Table 3:** Regression models of the association between handgrip strength or covariates and the indices of cognitive performance in adults with hypertension.

Variables		Q1	Q2 B (95% Confidence Interval)	Q3 B (95% Confidence Interval)	R2
Digit span forward (*n* = 3534)	Total	Reference	0.18 (0.07, 0.30) ***	0.20 (0.09, 0.32) ***	0.11
	Male	Reference	0.27 (0.14, 0.40) ***	0.31 (0.18, 0.44) ***	0.12
	Female	Reference	0.11 (−0.14,0.37)	−0.12 (−0.51, 0.28)	0.12
Digit span backward (*n* = 3530)	Total	Reference	0.01 (−0.09, 0.12)	−0.04 (−0.07, 0.15)	0.16
	Male	Reference	−0.05 (−0.17, 0.08)	−0.05 (−0.18, 0.07)	0.15
	Female	Reference	−0.12 (−0.35, 0.12)	−0.07 (−0.43, 0.30)	0.25
Verbal fluency (*n* = 3534)	Total	Reference	0.39 (−0.11, 0.79)	0.63 (0.22, 1.05) **	0.09
	Male	Reference	0.46 (−0.01, 0.93)	0.64 (0.16, 1.11) **	0.09
	Female	Reference	0.14 (−0.94, 0.67)	0.55 (−0.71, 1.84)	0.13
Delay recall (*n* = 3532)	Total	Reference	0.19 (0.01, 0.37) *	0.15 (−0.03, 0.33)	0.13
	Male	Reference	0.22 (0.01, 0.42) *	0.22 (0.01,0.42) *	0.13
	Female	Reference	0.27 (−0.14, 0.70)	0.31 (−0.32,0.95)	0.18

Note: Q1, Low tertile of relative handgrip strength (rHGS); Q2, Moderate tertile of relative handgrip strength (rHGS); Q3, High tertile of relative handgrip strength (rHGS). The adjusted model included age, sex, education years, setting, alcohol consumption, smoking, health condition, work physical activity, and leisure physical activity; *, *p* < 0.05, **, *p* < 0.01; ***, *p* < 0.001.

## Data Availability

The publicly archived datasets can be retrieved from http://www.who.int/healthinfo/sage/en accessed on 29 March 2021.
